# Time-dependent changes in hypoxia- and gliosis-related factors in experimental diabetic retinopathy

**DOI:** 10.1038/s41433-018-0268-z

**Published:** 2018-11-06

**Authors:** Limin Gu, Hua Xu, Chaoyang Zhang, Qian Yang, Limei Zhang, Jingfa Zhang

**Affiliations:** 1 Department of Ophthalmology, Third Affiliated Hospital of Second Military Medical University, Shanghai, China; 2 Department of Ophthalmology of Shanghai Tenth People’s Hospital, Tongji Eye Institute, Tongji University School of Medicine, Shanghai, China; 3 Department of Ophthalmology, Children’s Hospital of Soochow University, Suzhou, China; 4 Shanghai Peace Eye Hospital, Shanghai, China; 5 Department of Ophthalmology, Renji Hospital, Shanghai Jiaotong University School of Medicine, Shanghai, China

## Abstract

Diabetes causes various biochemical changes in the retina; long-term changes in the factors associated with hypoxia and gliosis have rarely been reported. The present study was conducted to explore the changes in these factors in a time-dependent manner in experimental diabetic retinopathy (DR). Diabetes was induced in Sprague–Dawley rats by intraperitoneal injection of streptozotocin. The expression of the following factors was examined using immunofluorescence and western blot analysis at 0.5, 1, 2, 4 and 6 months after diabetes onset: hypoxia-inducible factor-1alpha (HIF-1alpha), vascular endothelial growth factor (VEGF), erythropoietin (EPO), erythropoietin receptor (EPOR), glial fibrillary acidic protein (GFAP), vimentin, glutamate-aspartate transporter (GLAST) and glutamine synthase (GS). The expression of factors such as HIF-1alpha, VEGF, EPO, EPOR, GFAP and vimentin, was up-regulated with the progression of diabetes in the diabetic rat retinas compared to the expression in normal control retinas, whereas the expression of GS and GLAST was down-regulated. Changes in EPO and EPOR appeared 2 weeks after diabetes onset. HIF-1alpha, VEGF and GFAP started to increase at 1 month and vimentin at 4 months after diabetes onset. GS and GLAST started to decrease at 1 month after diabetes onset. The expression of these factors, which are involved in the processes of hypoxia and gliosis, varied at different stages of DR. The time-course change may be helpful in the evaluation of the progression of DR, and it may indicate the optimal intervention time points for DR.

## Introduction

Diabetic retinopathy (DR) remains a major complication of diabetes and a leading cause of blindness among adults worldwide. In DR, microangiopathy, neurodegeneration, neuroinflammation and dysregulation of neurovascular cross-talk are responsible for the progression of both early non-proliferative DR and advanced proliferative DR [1, 2].

The exact mechanisms of DR development are not entirely clear. Studies have shown that diabetes affects all major cell types in the retina [3]. Most researchers agree that hyperglycaemia is the initial cause of retinal damage. Hyperglycaemia induces abnormal functioning of the mitochondria, up-regulates the production of excess reactive oxygen species, injures vascular endothelial cells and increases inflammatory factors. These changes promote leukostasis and micro-thrombosis formation, which induce local capillary occlusion resulting in retinal hypoxia; this leads to increased expression of hypoxia-inducible factor-1alpha (HIF-1alpha), vascular endothelial growth factor (VEGF), erythropoietin (EPO) and EPO receptor (EPOR) [4]. VEGF is the main factor causing breakdown of the blood–retinal barrier (BRB) and retinal neovascularisation, which lead to diabetic macular oedema (DME) and proliferative DR [5, 6]. Anti-VEGF therapy has become popular in the treatment of different ophthalmic diseases, such as proliferative DR [7], DME [8], neovascular age-related macular degeneration [9], retinopathy of prematurity [10] and neovascular glaucoma [11]. However, there are concerns regarding the effectiveness and safety of long-term anti-VEGF therapies [12]. EPO, a two-edged sword in the progression of DR [13], has been used in the treatment of different ophthalmic conditions with promising results [14]. EPO is reportedly involved in ophthalmic neuroprotection [15], neurotrophism [16], angiogenesis [17, 18] and may have anti-inflammatory effects [19]. In one study, early retinal EPO suppression maintained retinal vascular stability, whereas late supplementation seemed to contribute to neovascularisation in the mouse model of oxygen-induced retinopathy. Therefore, understanding the role(s) of EPO in ophthalmic conditions and the timing of EPO intervention in patients are of great importance [18].

There is increasing evidence that shows that retinal neuronal dysfunction occurs early in DR and may even precede BRB breakdown [20]. It is generally viewed that neuronal apoptosis shares the same mechanisms with vascular damage, which involve inflammation, oxidative stress and hypoxia. In addition, other mechanisms are also involved, such as glutamate excitotoxicity, which is regulated by glial cells, especially Müller cells. Müller cells undergo reactive gliosis following acute retinal injury, chronic neuronal stress or diabetes onset [21]; reactive gliosis is characterised by changes in the cell shape due to alterations in intermediate filament production [22], which include an increase in the expression of glial fibrillary acidic protein (GFAP) [23, 24] and vimentin [25]. The impact of diabetes on Müller cells in the early stage of the DR includes the dysregulation of glutamate metabolism due to the down-regulation of glutamine synthetase (GS) and glutamate-aspartate transporter (GLAST), which results in glutamate accumulation, leading to retinal neurotoxicity [26, 27]. However, the changes in these cytokines/factors during DR progression have not been clearly illustrated, and this causes difficulties in the precise pharmacological treatment of DR.

In the present study, we examined the time-course changes in hypoxia-related molecules (such as HIF-1alpha, VEGF, EPO and EPOR) and glial cell-related proteins (such as GS, GLAST, GFAP and vimentin) in streptozotocin (STZ)-induced diabetic rats to elucidate the disease development spectrum and provide an optimal time point for DR treatment.

## Materials and methods

### Animals

Male Sprague–Dawley rats were randomly divided into two groups: a normal control and a diabetic group. Rats weighing approximately 180 g were purchased from Slaccas, SIBS, Shanghai, China. The animals were treated in accordance with the ARVO Resolution on the Care and Use of Laboratory Animals. All rats were housed under a normal 12-h light/dark schedule with ad libitum access to food and water. Diabetes was induced by intraperitoneal injection of STZ (60 mg/kg [of body weight] freshly dissolved in citric buffer, pH 4.5); diabetes was confirmed by a blood glucose level exceeding 250 mg/dL for three consecutive days. We measured the blood glucose level at the following time points: 1 week, 2 weeks (D2w), 1 month (D1m), 2 months (D2m), 4 months (D4m) and 6 months (D6m) after streptozotocin administration. The normal control rats were injected with an equal volume of citric-acid buffer.

The rats were killed at the following time points: D2w, D1m, D2m, D4m and D6m after diabetes onset. Both eyes were enucleated immediately for the studies described below.

### Western blot analysis

The retinas were lysed in a RIPA buffer (Beyotime Institute of Biotechnology, China) for protein extraction. The protein concentration was determined using the bicinchoninic acid protein assay kit (Thermo Scientific, Rockford, IL, USA). For the western blot analysis, 40 μg of the protein was dissolved in sodium dodecyl sulfatepolyacrylamide gels (10%) and transferred electrophoretically onto a nitrocellulose membrane (Bio-Rad, Hercules, CA, USA). The membranes were cut into several blots based on the size of the detected proteins and were blocked with 5% nonfat milk in TBST (50 mM Tris, pH 7.6; 0.9% NaCl; and 0.1% Tween-20) for 1 h at room temperature. Then, the blots were separately incubated overnight at 4 °C with the primary antibodies, i.e., HIF-1alpha (1:500, MAB5382, Rolling Meadows, IL, USA), VEGF (1:500, SC152, Santa Cruz Biotechnology, Santa Cruz, CA, USA), EPO (1:500, ab65394, Abcam, Cambridge, MA, USA), EPOR (1:500, SC679, Santa Cruz Biotechnology), GFAP (1:500, ab7260, Abcam), vimentin (1:500, 3634-100, BioVision, Milpitas, CA, USA), GLAST (1:500, ab416, Abcam) and GS (1:1,000, ab64613, Abcam). After washing thrice for 5 min each time, the membranes were incubated with the respective secondary antibodies, e.g., anti-mouse IgG (1:5,000, 610-431-002, Rockland, Pottstown, PA, USA) and anti-rabbit IgG (1:5,000, 611-131-002, Rockland) in 0.1% phosphate-buffered saline (PBS)-T with 5% low-fat milk (Guangming Company, Shanghai, China) for 1 h at room temperature. After extensive washing, we examined the blots using the Odyssey infra-red imaging system (LI-COR Biosciences, Lincoln, NE, USA). The densitometric values for the proteins of interest were normalized using beta-actin (1:500, A5441, Sigma-Aldrich, St. Louis, MI, USA).

The number of eyes in each experimental group is denoted by *n*. To detect the expressions of VEGF, EPO, EPOR, GFAP, vimentin and GS, six independent experiments were performed (*n* = 6); for GLAST, *n* = 5; for HIF-1alpha, *n* = 9. For each independent experiment, one retina was used from each group.

### Immunofluorescence

The eyeballs were fixed at 4 °C in PBS-buffered 4% paraformaldehyde for 24 h, and the anterior segment of the eyeball, including the cornea, iris and lens, was dissected under microscopy. The rest of the eyecup was dehydrated in 30% sucrose solution for 2 h and embedded in optimal cutting temperature compound for cryosectioning. Sections (10 μm) were cut on a Leica microtome (German) and mounted on adhesion microscope slides (Citoglas Company, Taizhou, China). Slides with sections were dried for 4 h at room temperature.

For immunostaining, the sections were incubated in PBS for 10 min; then, they were permeabilised in 0.25% Triton X-100 for 10 min. After washing thrice in PBS, the sections were blocked with 1% BSA for 30 min; then, they were incubated overnight at 4 °C with primary antibodies, i.e., HIF-1alpha (1:100, MAB5382, Chemicon, USA), Flk-1 (SC-101820, Santa Cruz Biotechnology), VEGF (1:100, SC-152, Santa Cruz Biotechnology), EPO (1:500, ab65394, Abcam), GFAP (1:500, ab7260, Abcam) and GS (1:500, ab64613, Abcam). The sections without primary antibody served as negative control. After being washed for 15 min in PBS, the sections were incubated with the appropriate secondary antibody (1:100, anti-mouse FITC or anti-mouse CY3) for 1 h at room temperature in the dark. Then, the sections were coverslipped for examination under a fluorescent microscope.

### Statistical analysis

The data are presented as means ± standard error (SE) from at least six independent experiments. The statistical analysis was performed using one-way ANOVA with Dunnett’s post-test (SPSS software, version 22.0, IBM Corp., Armonk, NY, USA). A *p* value of 0.05 or less was considered statistically significant.

## Results

### The up-regulation of EPO and EPOR with diabetic retinopathy progression

**Fig. 1:**
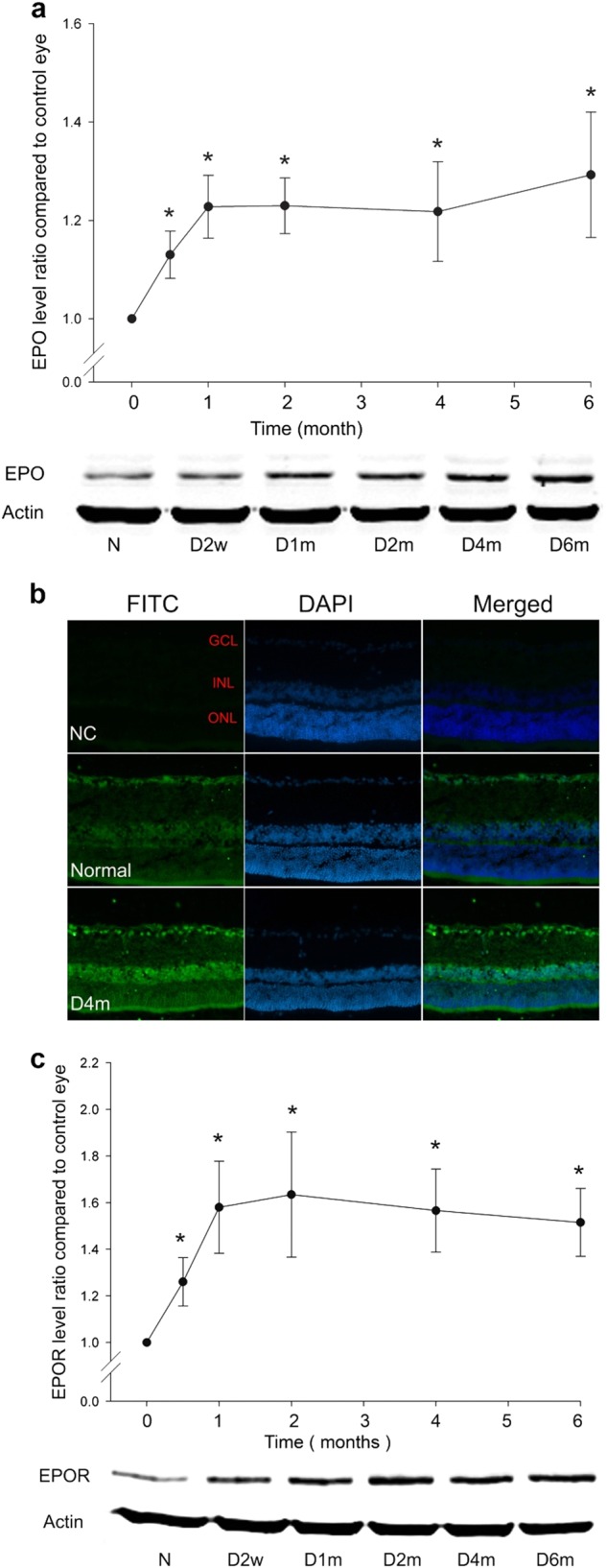
The protein expressions of EPO and EPOR with the progression of diabetes. **a** The time-dependent expression of EPO in the diabetic rat retinas from 2 weeks to 6 months. *n* = 6, and * denotes *p* < 0.05 compared with the normal control group. **b** Immunostaining of EPO in both normal control and diabetic rat retinas (magnification: ×200). **c** The time-dependent expression of EPOR with the progression of diabetes from 2 weeks to 6 months. *n* = 6 and * denotes *p* < 0.05 compared with the normal control group. EPO erythropoietin, EPOR erythropoietin receptor

When compared with that in the normal control group, the protein expression of EPO and EPOR in the retina in the diabetic group was up-regulated with the progression of diabetes. The expression of EPO in the diabetic group was about 1.13-fold (at 2 weeks, *n* = 6, *p* = 0.022) and 1.3-fold (at 6 months, *n* = 6, *p* = 0.012) of  that in the normal control group (Fig. 1a). The immunostaining of EPO confirmed the above changes; EPO was detected mainly in the ganglion cell layer (GCL) and inner nuclear layer (INL) in the normal control group and it was increased in the retinas of the diabetic group (Fig. 1b). The expression of EPOR was also significantly increased 2 weeks after diabetes onset, and it reached a plateau after 1 month. In comparison with that in the normal control group, the expression of EPOR in the diabetic group was approximately 1.26-fold (at 2 weeks, *n* = 6, *p* = 0.031) and 1.5-fold (at 1 month, *n* = 6, *p* = 0.006) (Fig. 1c).

### The up-regulation of HIF-1alpha and VEGF with diabetic retinopathy progression

**Fig. 2:**
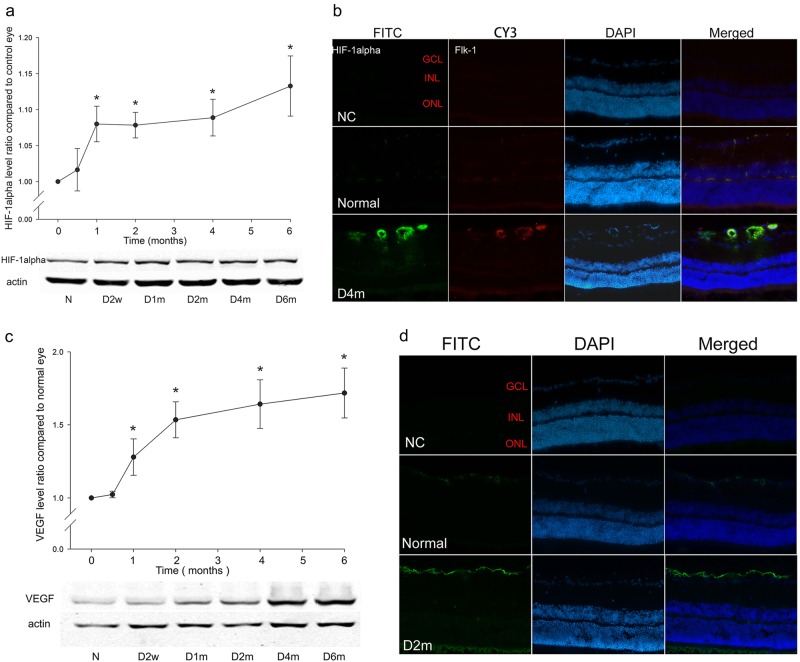
The expressions of HIF-1alpha and VEGF protein with the progression of diabetes. **a** The expression of HIF-1alpha protein time-dependently increased from 2 weeks to 6 months after diabetes onset. *n* = 9, and * denotes *p* < 0.05 compared with the normal control group. **b** Double staining of HIF-1alpha (FITC) and Flk-1 (CY3) in both normal control and 4-month diabetic rat retinas (magnification: ×200). **c** The time-dependent expression of VEGF detected with western blot analysis in diabetic rat retinas from  2 weeks to 6 months. *n* = 6, and * denotes *p* < 0.05 compared with the normal control group. **d** The immunostaining of VEGF in both the normal control and 2-month diabetic rat retinas (magnification: ×200)

The expression of HIF-1alpha in the diabetic group was increased 2 weeks after diabetes onset and it was maintained at low-to-moderate levels from 1 to 6 months. The expression of HIF-1alpha in the retinas of the diabetic group was approximately 1.08-fold (at 1 month), 1.09-fold (at 2 months), 1.11-fold (at 4 months) and 1.14-fold (at 6 months) of  that in the normal control group (Fig. 2a, *n* = 9, *p* < 0.05). Immunofluorescence showed mild HIF-1alpha staining in the normal control group, whereas there was enhanced staining in the 4-month diabetic retinas; the staining was mainly confined to the area of retinal vasculature (co-localisation with Flk-1, an endothelial cell marker) (Fig. 2b).

For the downstream targets of HIF-1alpha, the expression of VEGF was increased with diabetes progression. The expression of VEGF in the diabetic group was approximately 1.3-fold (at 1 month) and 1.7-fold (at 6 months) of  that in the normal control group (Fig. 2c, *n* = 6, *p* < 0.05). To validate these changes in VEGF expression, immunofluorescence was performed. As shown in Fig. 2d, VEGF immunostaining was mainly detected in the GCL in the normal control group, whereas in the 2-month diabetic rat retinas, the immunostaining of VEGF in the GCL was significantly increased (Fig. 2d).

### The expressions of GS and GLAST decreased with diabetic retinopathy progression

**Fig. 3:**
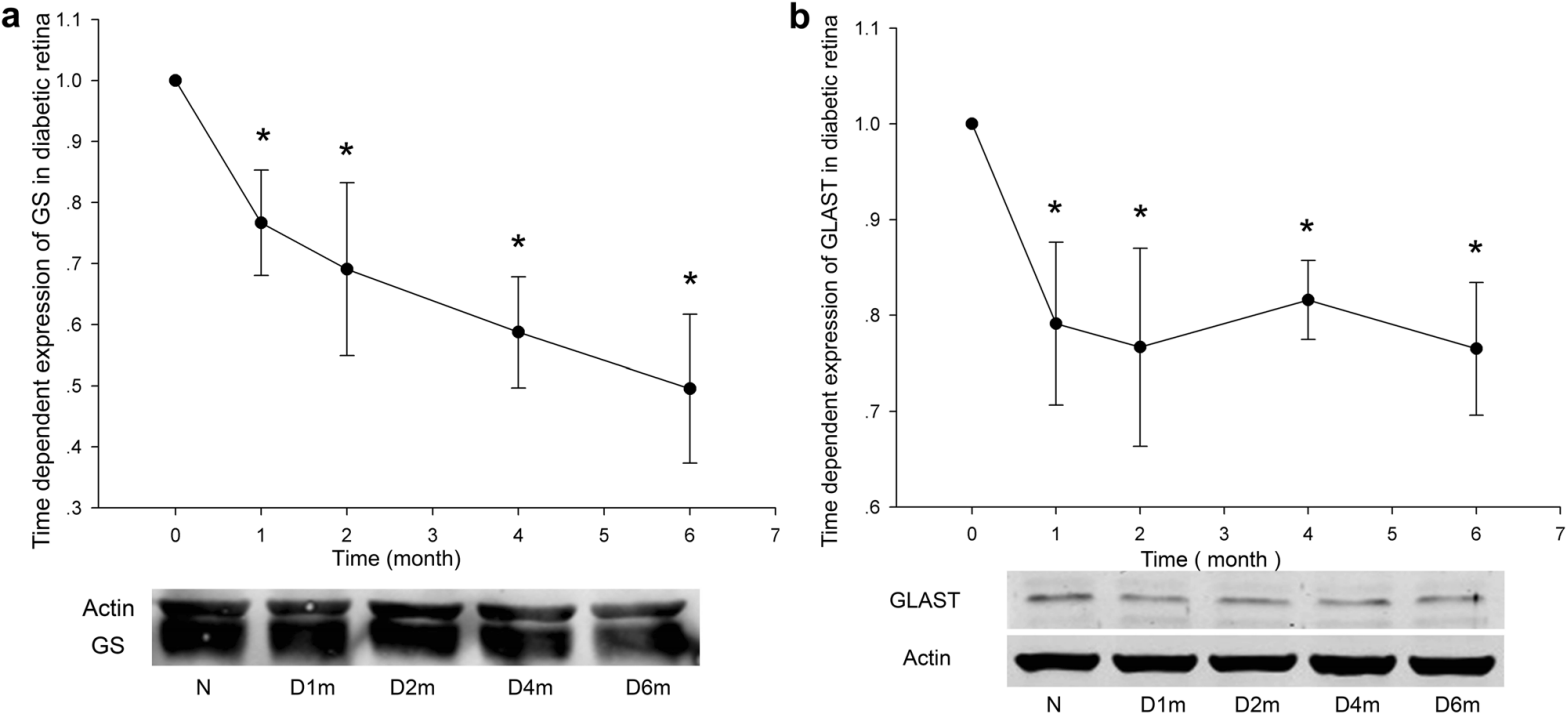
The expressions of GS and GLAST with the progression of diabetes. The time-dependent expressions of GS (**a**) and GLAST (**b**) were detected with western blot analysis in diabetic rat retinas from 1 month to 6 months. *n* = 6 (a), n=5 (b),  and * denotes *p* < 0.05 compared with the normal control group

To test the changes in the glial cells, especially in Müller cells, in the diabetic retina, the expression of GS and GLAST was examined. With increasing duration of hyperglycaemia, the expression of GS and GLAST gradually decreased. Retinal GS expression in the diabetic group decreased by 23% (at 1 month, *n* = 6, *p* = 0.038) and by 51% (at 6 months, *n* = 6 , *p* = 0.0078) compared with that in the normal control group (Fig. 3a). Retinal GLAST expression in the diabetic group decreased by 23% (at 1 month, *n* =5 , *p* = 0.015) and by 24% (at 6 months, *n* = 5, *p* = 0.0027) compared with that in the normal control group (Fig. 3b). The gradual decreases in GS and GLAST expressions can contribute to the accumulation of glutamate in diabetic retinas and cause neuronal death.

### The expressions of GFAP and vimentin increased with diabetes progression

**Fig. 4:**
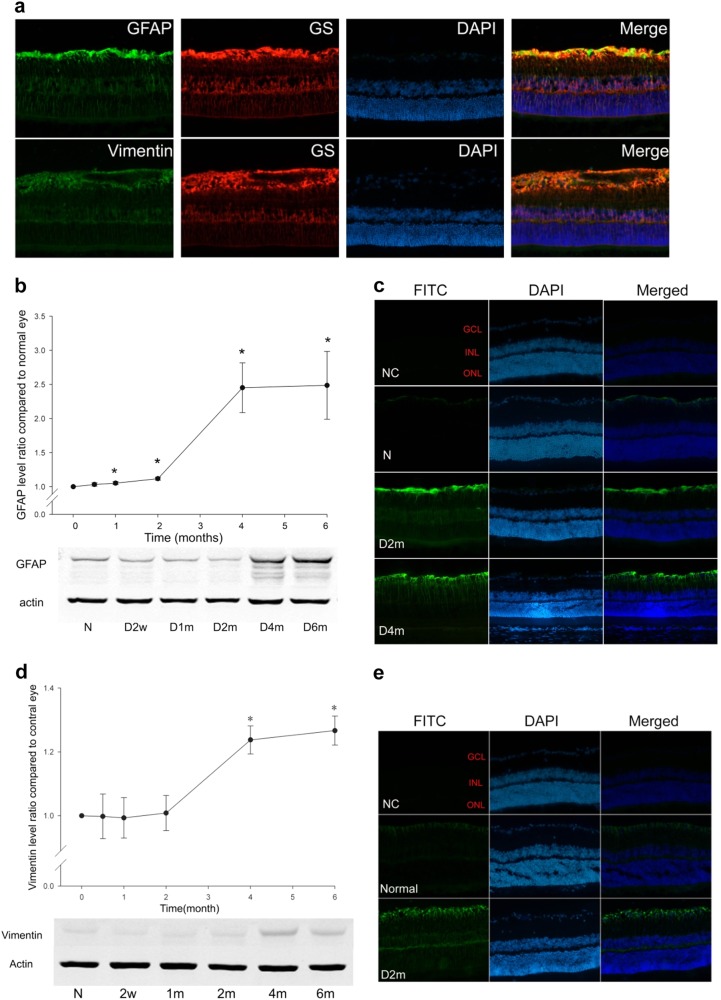
The expressions of GFAP and vimentin with the progression of diabetes. **a** GS co-staining with GFAP or vimentin in the 4-month diabetic rat retinas (magnification: ×200). **b** The time-dependent expression of GFAP was detected with western blot analysis in diabetic rat retinas from 2 weeks to 6 months. *n* = 6, and * denotes *p* < 0.05 compared with the normal control group. **c** Immunostaining of GFAP in normal control and diabetic rat retinas (magnification: ×200). **d** The time-dependent expression of vimentin was detected with western blot analysis in the diabetic rat retinas from 2 weeks to 6 months. *n* = 6 and * denotes *p* < 0.05 compared with the normal control group. **e** Immunostaining of vimentin in both the normal control and diabetic rat retinas (magnification: ×200)

Intermediate filament proteins (GFAP and vimentin) are the glial cell markers that are mainly expressed by astrocytes and Müller cells in the retina. To confirm cell-specific increases in GFAP and vimentin expressions, we co-localised both markers with GS, which is specific for Müller cells. The immunofluorescence result showed that both GFAP and vimentin were co-localised with GS in 4-month diabetic rat retinas (Fig. 4a), indicating the activation of Müller cells in diabetes.

The level of GFAP expression in the 1-month diabetic rat retina was approximately 1.05-fold of  that in the normal retina (Fig. 4b, *n* = 6, *p* = 0.014); the level reached a plateau at 4 months (*n* = 6, *p* = 0.001) and was approximately 2.5-fold of that in the normal control group. The immunostaining showed that GFAP was mainly expressed in the nerve fibre layer (NFL) and GCL. With diabetes progression, the expression of GFAP was significantly increased in Müller cells (Fig. 4c).

The expression of vimentin was also increased with increasing duration of diabetes; it was approximately 1.23-fold (at 4 months, *n* = 6, *p* = 0.0001) and 1.26-fold (at 6 months, *n* = 6, *p* = 0.0001) of that in the normal control group (Fig. 4d). In the normal control group, vimentin was mainly expressed in the NFL and GCL, and it was significantly increased in the diabetic group (Fig. 4e).

## Discussion

The mechanisms leading to the development of DR are indeed complex. Evidence shows that DR involves all major cell types of the retina [28]. Multiple interactive mechanisms may come into play resulting in cellular damage and the development of this devastating complication [29, 30]. Our results showed that both hypoxia and gliosis are involved in DR. Although the relationship and interaction of these factors have not been completely elucidated, the present study shows there are time-dependent changes in both hypoxic- and gliosis-related factors, and these changes may indicate the optimal time for intervention or treatment of DR.

Hypoxia is commonly considered to be the central pathogenic stimulus for DR. All hypoxia-dependent events in cells seem to share a common denominator: HIF-1, which is composed of both alpha and beta subunits. The beta subunit of HIF-1 is constitutively expressed, whereas the stability and transcriptional activity of the alpha subunit is precisely controlled by intracellular oxygen concentration. Under normoxia, the level of HIF-1alpha protein is quite low due to rapid ubiquitination and subsequent proteasomal degradation; however, under hypoxia, the above process is suppressed, resulting in HIF-1alpha accumulation and formation of the active complex with HIF-1beta [31]. Under hypoxic conditions, HIF-1 triggers the activation of more than 100 downstream genes identified with varying functions, including VEGF, EPO and EPOR, which are associated with angiogenesis [32–34]. HIF-1alpha, as an essential transcription factor mediating the adaptation of cells to low-oxygen tensions, is precisely regulated by hypoxia and hyperglycaemia, which are major determinants of the chronic complications associated with diabetes. Apart from hypoxia, glucose also affects the expression and activation of HIF-1alpha. In fact, the relationship between glucose and HIF-1alpha is sometimes mutual [35, 36]. HIF-1alpha up-regulates the expression of nearly all enzymes involved in the process of glycolysis as well as glucose transporter 1 (GLUT1) and GLUT3 mediating cellular glucose uptake [37]. High glucose could also up-regulate the protein level of HIF-1alpha and increase its transcriptional activity both in vitro and in vivo [38, 39]. Therefore, the contribution of high glucose inducing the up-regulation of HIF-1alpha cannot be excluded. Although hyperglycaemia can up-regulate HIF-1alpha signalling in some specific cell types, the effect of hyperglycaemia on HIF-1alpha remains controversial; the inhibition of HIF-1alpha by high glucose has also been suggested [40]. In this study, we found that HIF-1alpha expression was moderately increased by 1.02-fold (*n* = 9, *p* = 0.856) 2 weeks after diabetes onset, which became significant at 1.08-fold (*n* = 9, *p* = 0.026) 4 weeks after diabetes onset, compared to that of the normal control group. Due to the complex interplay between HIF-1alpha expression and activation and diabetes as well as hyperglycaemia, the exact mechanism for HIF-1alpha up-regulation under diabetic conditions needs further exploration.

In this study, we investigated the expressions of these proteins and found that HIF-1alpha was specifically localised in the area of retinal vascular endothelial cells in diabetic rat retinas. This finding was consistent with those of previous reports showing that hypoxia induces a significant increase in HIF-1alpha protein expression in vascular endothelial cells both in vitro [41, 42] and in vivo [43]. Further evidence, supporting that HIF-1alpha is associated with diabetes, comes from a study by Chavez et al. [44], who demonstrated a transient increase in the expression of HIF-1alpha in nerves that peaked between 4 and 6 weeks and declined 8 weeks after induction of experimental diabetes in rats.

For the downstream targets of HIF-1, such as VEGF and EPO, our findings are consistent with those of published studies [19, 45–47]. VEGF has long been known to contribute to angiogenesis and increase the permeability of the vascular endothelium. Anti-VEGF therapy aimed at preventing macular oedema and retinal neovascularisation has been widely used, and it has produced satisfactory results [48–51]. EPO is still being considered a “two-faced Janus” factor in the retina where it acts by binding to a trans-membrane receptor (EPOR). EPO has an angiogenic activity similar to that of VEGF, i.e., stimulation of proliferation, migration and angiogenesis in the endothelial cells that express EPOR [52–55]. Over the past years, many studies have suggested that EPO has a neuroprotective role in the retina. With regard to DR, EPO is indeed a pathogenic factor in proliferative DR, but it also plays a protective role in early DR [15, 56, 57]. Therefore, the timing for intervention with EPO is important in the treatment of DR. In addition to EPO, which is produced locally in the retina, EPOR is also expressed in the retina [58]. The exact sites of EPO and EPOR expression in the retina, e.g., cell-specific expression in the retina, have not yet been defined with certainty. Our study showed that EPO is extensively expressed in the diabetic rat retina, especially in the GCL, INL and outer nuclear layer. However, immunostaining showed a relatively low level of expression of EPOR, which could not be detected in the normal or diabetic rat retinas by immunofluorescence.

Müller cells play a pivotal role in the development of DR. Early DR pathology is often accompanied by increased expression of GFAP, which is a sensitive non-specific biomarker of the response to retinal injuries and diseases [59, 60]. Previous studies indicated that Müller cells were a source of retinal VEGF in diabetic rats and that VEGF derived from Müller cells played an important role in retinal inflammation in DR [61].

The results of our experiment revealed that both GLAST and GS were down-regulated in the retina with diabetes progression. This finding may provide new insight into the putative mechanisms of neuronal apoptosis in DR. For example, the decreased GLAST and GS expressions in Müller cells lead to accumulation of glutamate in the extracellular space; thus, causing retinal neuronal cell death [62, 63]. Dysfunction of glutamate metabolism in Müller cells is one of the earliest diabetes-induced changes in the retina. This change precedes the increase in GFAP expression that is up-regulated after a few weeks of experimental diabetes onset [23, 64]. Thus, GLAST and GS in Müller cells appear to be the molecules that are particularly vulnerable in the early course of diabetes.

Because of multifactorial contributions to pathogenesis, as well as their chronology and interplay, it is difficult to understand the mechanisms of DR completely. Investigations of the aetiology of DR will require genetic dissection of the different molecular pathways in rodents, determination of the interrelationships between vascular and neuronal cells, and proof-of-principle studies in large animal models [65]. In the present study, we examined the time-course changes in hypoxia-related molecules (such as HIF-1alpha, VEGF, EPO and EPOR) and glial cell-related proteins (such as GS, GLAST, GFAP and vimentin) in STZ-induced diabetic rats to elucidate the disease development spectrum and provided time-based information for DR progression. It is crucial to identify an upstream regulator of the multiple pathogenic factors not only to improve understanding of how DR develops, but also to uncover new potential therapeutic targets.

### Summary

#### What was known before

- Diabetes causes various biochemical changes in the retina; long-term changes in the factors associated with hypoxia and gliosis have rarely been reported.

#### What this study adds

- The results of our experiment revealed that both GLAST and GS were down-regulated in the retina with diabetes progression.
- This finding may provide new insight into the putative mechanisms of neuronal apoptosis in DR.
